# Use of antibiotics in poultry and poultry farmers- a cross-sectional survey in Pakistan

**DOI:** 10.3389/fpubh.2023.1154668

**Published:** 2023-07-11

**Authors:** Um e Habiba, Amjad Khan, Elia John Mmbaga, Ivan Robert Green, Muhammad Asaduzzaman

**Affiliations:** ^1^Department of Community Medicine and Global Health, Institute of Health and Society, Faculty of Medicine, University of Oslo, Oslo, Norway; ^2^Department of Pharmacy, Quaid-i-Azam University, Islamabad, Pakistan; ^3^Department of Chemistry and Polymer Science, Stellenbosch University, Stellenbosch, South Africa

**Keywords:** antimicrobial resistance, poultry, poultry farmers, antibiotic use, Pakistan, food safety, one health, poultry farming practices

## Abstract

**Background:**

Antimicrobial resistance (AMR) which has been ascribed to be due to community carriage of antibiotic-resistant bacteria is highly prevalent in the WHO South-East Asia region. One of the major reasons for this is the misuse of antibiotics in animal farming practices and at the community level, which threatens both human and animal health. However, this problem of antibiotic misuse in poultry farms and in respective farmers is not well studied in countries like Pakistan.

**Methods:**

We conducted a cross-sectional study in rural Punjab to explore the current practices of antibiotic use in poultry and poultry farmers, associated factors, their healthcare-seeking behavior and biosecurity practices.

**Results:**

In the context of antibiotic use for poultry, 60% comprised of Colistin sulfate and Amoxicillin trihydrate whereas Colistin is considered as the last resort antibiotic. In addition, the significant consumption of antibiotics in poultry farms (60%) and poultry farmers (50%) was without prescription by either human health physicians or veterinarians. Most of the farms (85%) had no wastewater drainage system, which resulted in the direct shedding of poultry waste and antibiotic residue into the surrounding environment. The lack of farmers’ education, professional farm training and farming experience were the most significant factors associated with antibiotic use and knowledge of AMR.

**Conclusion:**

Our study findings show that it is necessary for an integrated AMR policy with the inclusion of all poultry farmers to be educated, a mass awareness program to be undertaken and that strict antibiotic usage guidelines be available to them. Such initiatives are also important to ensure food safety and farm biosecurity practices.

## Introduction

As per WHO misuse of antibiotic referred to buy antibiotics for animal and human use without prescription, takin antibiotics for viral infections, e.g., cold, flu and using antibiotics for growth promotion on farms (1).

Misuse of antibiotics in food-producing animal farming practices has become an inevitable challenge to the containment of global antimicrobial resistance (AMR) both in humans and in animals, particularly in low and middle-income countries (LMICs) (2). AMR has been gradually increasing over the last few decades, and currently, it accounts for almost 7 million deaths per year, which is estimated to increase to 10 million by the year 2050; with 90% of these deaths in LMICs of Africa and Asia (3, 4). Inadequate policies and regulations in LMICs have led to an increase in antibiotic consumption and subsequent drug-resistant infections to a great extent (5). Antibiotic use (in the human and animal sectors has the potential for transmission of AMR), encompassing the environment as well. This transmission occurs through direct contact between animals and humans as well as through food or shared environmental sources (6).

Antibiotic use in food animals started in the 1940s when the use of tetracycline in animals resulted in improved growth (7). Intensive use of antibiotics in food-producing animals has increased over the last decades because of the high demand for meat (8). According to a report presented by the Food and Agriculture Organization (FAO) and the Organization for Economic Co-operation and Development (OECD), the estimated poultry meat production in 2014 was 108.5 million tons, while in 2023, it is projected to reach 134.5 million tons. This inevitably puts increased pressure on farmers to produce more meat in the minimum time, e.g., 6 weeks instead of 9 or 10 weeks (9). Undoubtedly, more antibiotic residues exist in poultry production with no or negligible withdrawal periods. If the antibiotics are administered in food animals beyond the permissible limits and without adherence to the withdrawal period, this will be hazardous for human health (e.g., allergic reactions, AMR, and imbalance of intestinal microbiota) as well when they consume the meat and meat products (9). Changes in human microbiota along with the transmission of resistant genes eventually decreases the effectiveness of antibiotics used by that individual (9). Even farmers working in the poultry production facilities may have high rates of AMR due to occupational exposure (10).

Pakistan is among the top 10 countries that are producing food animals through modern farming practices and rely on antibiotics as growth promoters and for disease prevention (11). However, there is unfortunately no estimation of annual antibiotic use in food-producing animals in Pakistan. Thus, it is difficult to estimate the exact antibiotic usage for the treatment and prevention of diseases, and as growth promoters. More than 600,000 unqualified practitioners (locally known as quacks) are active for selling these antibiotics and roughly 50,000 unregistered products are available in local markets which exacerbates the situation further (12). While Pakistan is ranked as the third highest among LMICs for antibiotic consumption (13), it is a common practice there to seek treatment from a local medical store or use antibiotics by getting advice from relatives or through previous experience. Several studies have reported a high percentage (50% and above) for antibiotic prescriptions from local clinics (14, 15).

Apart from the direct effect of antibiotic use on AMR development in humans and animals, an abundance of resistant pathogens in the environment and elevated environmental pressure of them, are also major transmission factors in such circumstances. AMR transmission to the environment occurs in different ways, e.g., dissemination of animal waste (feces and urine, litter materials), uncontrolled grazing of animals, using organic fertilizer (animal waste), and the fact that pharmaceutical companies and municipalities dump their waste and human waste in the environment (16–18). In many LMICs including rural Pakistan, poultry wastes are ironically considered to be the best fertilizer for agricultural land. Antibiotics present in poultry wastes are mostly bioactive and result in increased antimicrobial resistance(AMR) in exposed bacteria in the surrounding environment (19). Therefore, the chances of resistant bacteria and gene transmission from poultry to human beings are high in rural areas because of shared living and sleeping areas with no proper waste disposal from poultry farms. Biosecurity measures are almost non-existent in small-scale farming in south Asia where poultry wastes are usually disposed into municipal drains or nearby open land (20).

While the burden of AMR is high and difficult to quantify in LMIC settings, there are multiple challenges to mitigate against it (21). Adequate knowledge about antibiotics, optimum biosecurity and prescription practices, and AMR awareness can play pivotal roles in the rational antibiotic use (22). For proper policy implementation, an understanding of the current poultry farming practices, the pattern of antibiotic use, and healthcare-seeking behavior for both farmers and farm animals are crucial. Therefore, in this study, we have focused on antibiotic use in commercial poultry farms and farmers along with their contributing factors in rural Pakistan.

## Materials and methods

### Study design, study area, and recruitment

**Figure 1:**
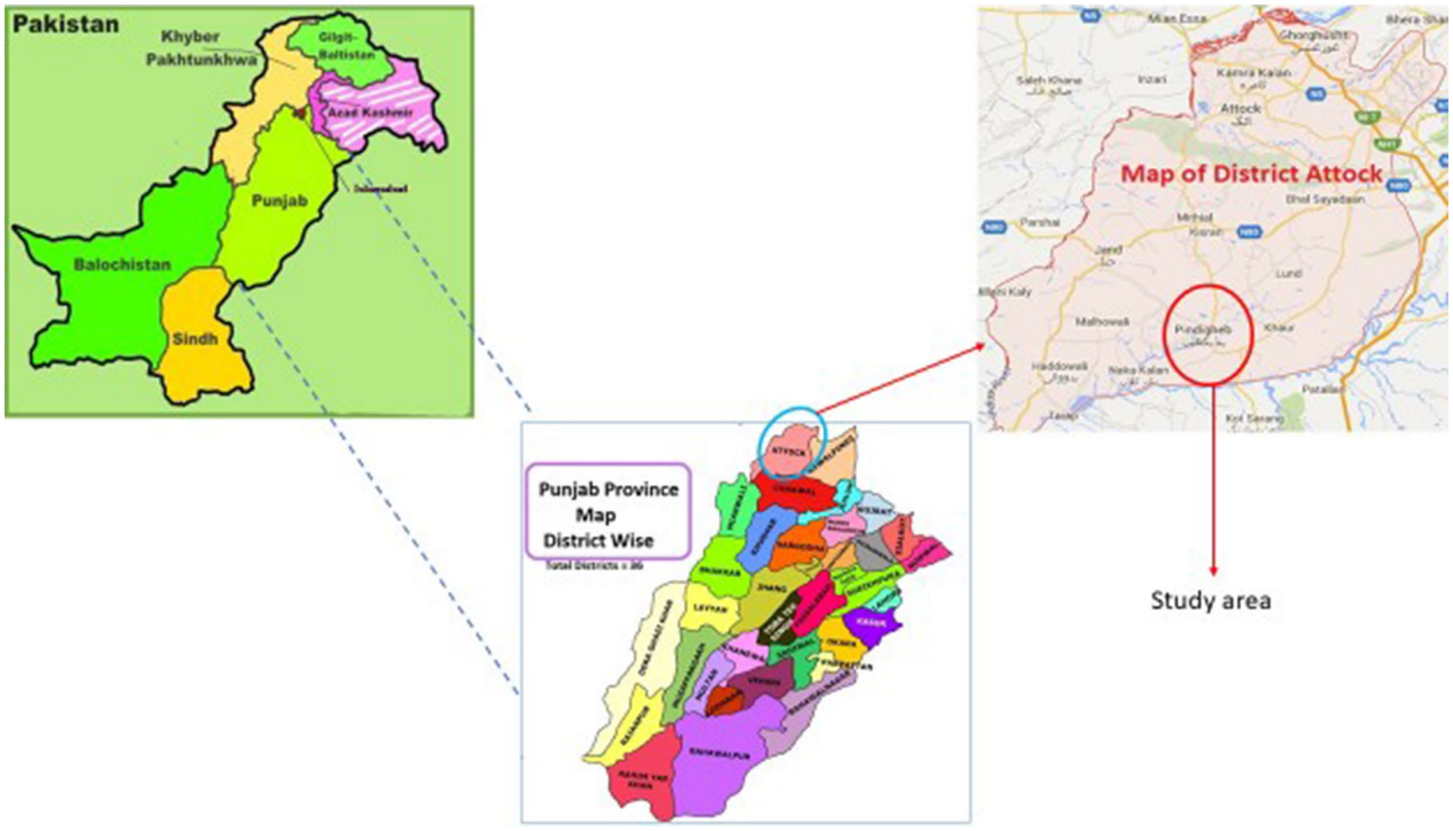
Map of study area (23, 24).

We conducted a cross-sectional survey for poultry farms and poultry farmers in rural Punjab, Pakistan from January to March 2021. The Tehsil (sub-district) named Pindi Gheb from Attock district in Punjab was selected as our study area which is one of the more densely populated districts in Punjab with a large number of poultry farms (Figure 1). From Tehsil Pindi Gheb, out of 134 villages, we randomly selected 10 as well as 4 farms per village (*n* = 40). The eligible participants were voluntarily agreed adult poultry farmers who provided their prior informed consent before data collection.

### Data collection

Data was collected using a validated and researcher administered questionnaire (which had been pre-tested in 4 non-study villages). Our questionnaire was adopted from a study conducted recently in North-western China regarding the use of antibiotics in poultry (25). For the use of antibiotics by farmers themselves a validated questionnaire from a study conducted in the Northwest region of Pakistan regarding self-medication and antibiotic use by the public (26). Questionnaire was comprised of 5 main sections: A structured questionnaire comprising of 5 main sections was used: (1) Characteristics of farms and demographic data of farmers. (2) Health care seeking behavior for antibiotic use in poultry farms. (3) Health care seeking behavior for antibiotic use by poultry farmers. (4) Disposal of poultry wastes. (5) Knowledge of poultry farmers about antibiotic use and resistance. The questionnaire was translated into Urdu to make it easy for farmers to understand and all the communication with farmers was in Urdu and Punjabi (local language). We also incorporated the suggestions and information from the local livestock officers, veterinary doctors, and medical doctors regarding the questionnaire development particularly to ask about illness and the use of antibiotics in both humans and poultry. All data were anonymized and entered in TSD (Services for sensitive data) provided by the University of Oslo.

### Statistical analysis

All statistical analyses were performed in an IBM SPSS Statistics version 28.0.1.1 (IBM Corp.) Descriptive statistic was used to analyze the characteristics of the study farms, demographics of farmers, and pattern of antibiotic use in poultry farms and farmers. Additionally, distributions of antibiotic misuse by the demographic characteristics and the education level of farmers were compared with the knowledge of farmers about antibiotics by cross-tabulation.

We also performed a chi-square test to check the association between the education level of participants and knowledge about antibiotics, AMR, and prohibited antibiotics in poultry. Statistical significance was considered at *p* < 0.05.

Our study variables include both dependent and independent variables. Dependent variable include purpose of antibiotics use while independent variables include education and professional farm training of farmers, knowledge of farmer about antibiotic resistance and prohibited antibiotics, physician prescription, veterinary doctor prescription, frequency of antibiotic, withdrawal time period follow up, method of disposing poultry waste.

We performed the regression analysis (binary and multinomial) for these dependent and independent variables but there was no statistical significance association between variables.

## Results

### Characteristics of the poultry farms and demographic data of farmers

A total of 40 poultry rearing farms and farmers were included in the study. All farmers were male. The duration in the poultry farming profession ranged from 2 months to 35 years but nearly two-thirds of the farmers (*n* = 25: 62.5%) had an experience of less than 15 years (Table 1).

**Table 1:** Characteristics of farms and demographic data of farmers (*N* = 40).

Characteristics	Total
	*n* (%)
No. of year/s in farming	
<15	25(62.5)
15–30	13(32.5)
>30	2(5.0)
Education level of farmers	
Not educated	10(25.0)
Primary	5(12.5)
Secondary	20(50.0)
Above secondary	5(12.5)
Professional farm training	
No	35 (87.5)
Yes	5(12.5)
Size of poultry farm	
Small (<2000 chickens)	15 (37.5)
Medium (2,000–4,000 chickens)	19(47.5)
Large (>4,000 chickens)	6(15.0)

Twenty farmers (50.0%) completed their secondary education (10 years of education) and 10 (25.0%) had no formal education. Thirty-five participants (87.5%) never attended any professional farm training. Nineteen poultry farms (47.5%) included in this survey were medium scale broiler farms which had 2000–4,000 chickens per farm (Table 1). The number of workers in the farms varied depending on the number of chickens. There was only one worker in all small-sized poultry farms having <2000 chickens.

### Health care-seeking behavior for antibiotic use in poultry farming

Our current study reveals an extensive use of antibiotics in all farms (*n* = 40: 100%), the major use of antibiotics as growth promoters (*n* = 18, 45%), lack of compliance (e.g., antibiotic administration in 50% of farms for only 1–3 days), and health care seeking from unqualified practitioners for antibiotics to a larger extent (*n* = 24, 60%) (Table 2). All participants reported using antibiotics in every flock and most of them (*n* = 33, 82.5%) reported the purchase of antibiotics from agents instead of pharmacy/drug stores. Agents act as a third party between the poultry farmers and feed/veterinary drug companies and supply feed and medicines to the poultry farms. Moreover, 45% (*n* = 18) of the respondents had received veterinary services from feed companies.

**Table 2:** Antibiotic use characteristics and healthcare-seeking behavior in Poultry farming (*N* = 40).

Variables	Total *N* (%)
Antibiotic/s use in poultry	
No	0(0)
Yes	40(100.0)
Veterinary doctor Prescription for getting antibiotic/s	
No	24(60.0)
Yes	16(40.0)
Source of veterinary services	
Local livestock officer	1(2.5)
Private veterinary doctor	14(35.0)
By Yourself	2(5.0)
Feed company	18(45.0)
Government source	5(12.5)
Source of getting antibiotic/s	
Agents	33(82.5)
Local pharmacy/drug shop	7(17.5)
Use of antibiotic/s for clinical conditions	
No	18(45.0)
Yes	22(55.0)
Use of antibiotic/s as Growth promotion	
No	22(55.0)
Yes	18(45.0)
Frequency of antibiotic/s use	
Occasionally*	11(27.5)
Regularly**	29(72.5)
No. of days of antibiotic/s administration	
1–3 days	20(50.0)
4–7 days	12(30.0)
>7 days	8(20.0)
Follow-up of withdrawal period	
No	19(47.5)
Yes	21(52.5)

*Occasionally: Have not used antibiotics in every flock. **Regularly: Used antibiotics in every flock.

**Figure 2:**
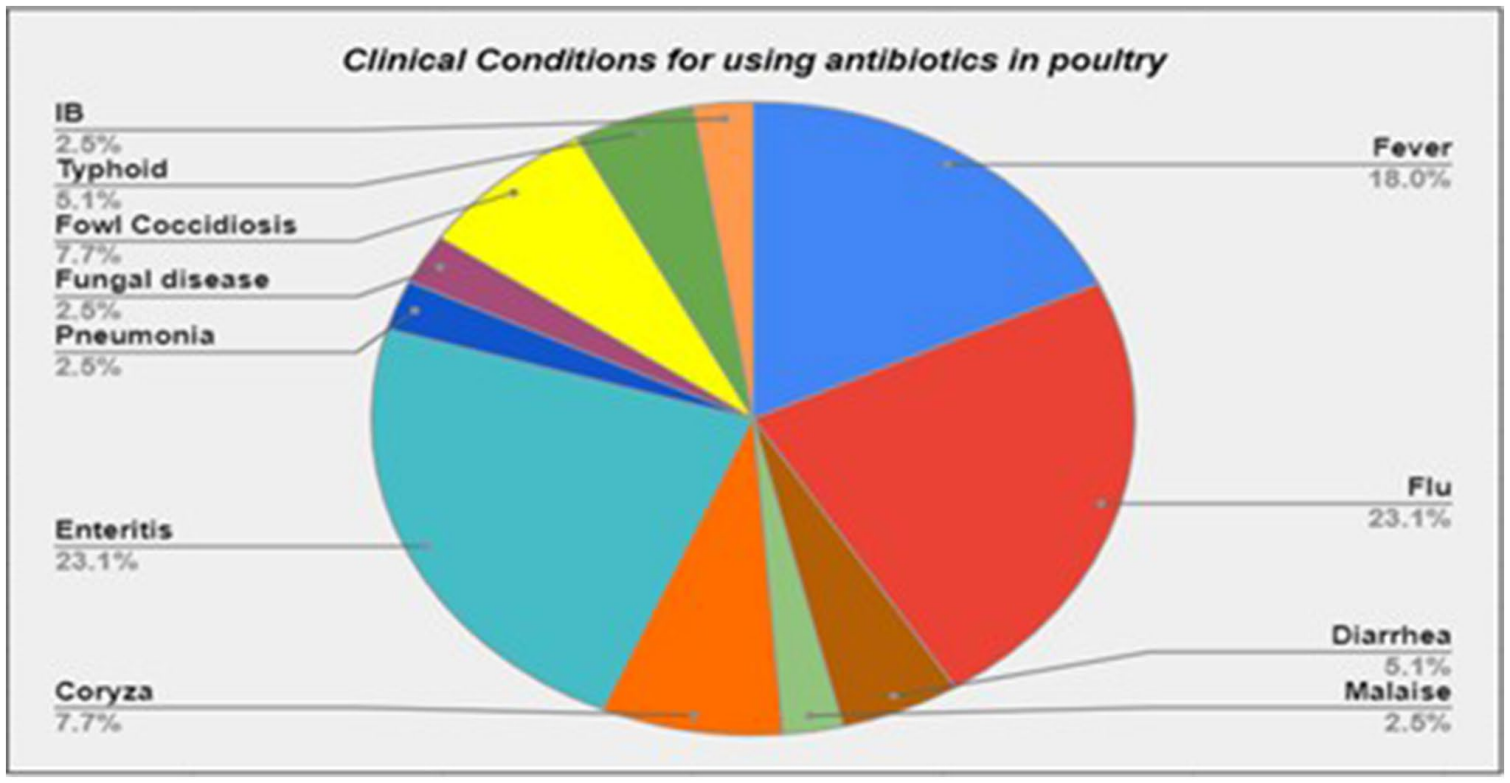
Clinical conditions for using antibiotics in poultry.

Furthermore, we found that about three-quarters of the participants (*n* = 29, 72.5%) frequently used antibiotics. Half of the poultry farmers (*n* = 21, 52.5%) did not follow withdrawal periods of the antibiotics. Interestingly, many of the farmers (*n* = 22, 55.0%) used antibiotics for clinical conditions, which did not require antibiotics, such as flu, fungal infections, or malaise (Figure 2).

### Pattern of antibiotic use in poultry farms and associated factors

Table 3 illustrates the pattern of antibiotic use including class and types. It can be noted that 12 classes of antibiotics, containing 18 types, were used in poultry farming by the participants in the study group. These antibiotics were used both separately and in combination with others. Out of these antibiotics, both colistin and a combination of colistin sulfate and Amoxicillin trihydrate were most frequently (*n* = 24, 60.0%) used followed by Enrofloxacin, Tylosin and Doxycycline (35.0, 25.0 and 22.5%) respectively. Apart from antibiotics, other antimicrobials, e.g., antivirals (Amantadine HCl) and antifungal (Nystatin) were used by 25.0 and 2.5% of poultry farmers for the treatment of viral and fungal diseases.

**Table 3:** Antibiotic classes and types used in commercial poultry farms.

Antibiotic class	Antibiotic	No of farms using antibiotics (N = 40)
		n (%)
Aminopenicillins	Amoxicillin trihydrate	3(7.5)
Tetracyclines	Chlortetracycline	1(2.5)
	Oxytetracycline	1(2.5)
	Doxycycline	9(22.5)
Polymyxins	Colistin	24(60.0)
Macrolides	Tylosin	10(25.0)
	Erythromycin	2(5.0)
Fluoroquinolones	Ciprofloxacin	1(2.5)
	Enrofloxacin	14(35.0)
Penicillin	Penicillin	2(5.0)
Polypeptides	Bacitracin	4(10.0)
Trimethoprim	Trimethoprim	1(2.5)
Sulfonamides	Sulfamethoxypyridazine	1(2.5)
	Sulfamethazine	1(2.5)
Aminoglycosides	Neomycin	4(10.0)
	Streptomycin	2(5.0)
	Gentamycin	2(5.0)
Nitrofurans derivatives	Furaltadone	1(2.5)
Aminopenicillins/polymyxins	Amoxicillin trihydrate + colistin sulfate	24(60.0)

As illustrated in Table 2, in 18 farms (45.0%), antibiotics were used as growth promoters. However, the pattern varied based on the farmers’ education level, professional farm training and health seeking behavior. As shown in Table 4, farmers having no education or primary level education used more antibiotics for growth promotion in poultry (*n* = 6, 60.0% and *n* = 4, 80.0%), as compared to those having secondary level education (*n* = 7, 35.0%) and above (*n* = 1, 20.0%). Likewise, professionally trained farmers had not used antibiotics as growth promoters contrary to those having no professional farm training (*n* = 18, 51.4%). The Chi-square test indicates the significant correlation between professional farm training and antibiotic use as a growth promoter (*p* = 0.05). While education level and antibiotic use as growth promoter had no significant correlation (*p* = 0.141).

**Table 4:** Associated factors related to use of antibiotics as growth promoters.

Farmers’ characteristics *n* (%)	Antibiotic/s used as growth promoter (18 out of 40)	Value of *p*
	*n* (%)	
Education level of farmers 40(100.0)		0.141
Not educated 10(25.0)	6(60.0)	
Primary 5(12.5)	4(80.0)	
Secondary 20(50.0)	7(35.0)	
Higher secondary or graduation 5(12.5)	1(20.0)	
Professional farm training		0.05
No 35 (87.5)	18(51.4)	
Yes 5(12.5)	0(0)	
Obtained antibiotics after prescription		0.436
No 24(60.0)	12(50.0%)	
Yes 16 (40.0)	6(37.5)	

Again, the majority (90.0%) of the respondents who were not educated had no knowledge about antibiotic usage and prohibited antibiotics in poultry; and no farmer in this category had knowledge about antibiotic resistance (Table 5). Farmers having a primary level of education had no knowledge about antibiotic usage, resistance, and prohibited antibiotics. Out of the 20 farmers who had a secondary level of education (*n* = 5, 25.0%), had a rudimentary knowledge about antibiotic usage (*n* = 3, 15.0%) had knowledge about prohibited antibiotics, and (*n* = 2, 10.0%) about antibiotic usage. The majority (4 out of 5, 80.0%) of the respondents having a higher secondary level of education or more, had some knowledge about antibiotic usage, while over half (*n* = 3, 60.0%) had knowledge about prohibited antibiotics and (*n* = 2, 40.0%) had knowledge about antibiotic resistance. There is a significant association between the education level of farmers and knowledge about antibiotic usage (*p* = 0.012) and prohibited antibiotics (*p* = 0.051).

**Table 5:** Knowledge of poultry farmers about antibiotics.

Variables	Total	Knowledge about AB use			Knowledge about prohibited AB			Knowledge about antimicrobial resistance		
	*N* (%)	No	Yes *N* (%)	Value of *p*	No	Yes	Value of *p*	No	Yes N (%)	Value of *p*
		*N* (%)			*N* (%)	*N* (%)		*N* (%)		
Education level of farmers				0.012			0.051			0.083
Not educated	10(25.0)	9(90.0)	1(10.0)		9(90.0)	1(10.0)		10(100)	0(0)	
Primary	5(12.5)	5(100.0)	0(0)		5(100.0)	0(0)		5(100)	0(0)	
Secondary	20(50.0)	15(75.0)	5(25.0)		17(85.0)	3(15.0)		18(90)	2(10.0)	
Higher secondary or graduation	5(12.5)	1(20.0)	4(80.0)		2(40,0)	3(60.0)		3(60)	2(40.0)	
Professional farm training				0.002			<0.001			0.017
No	35 (87.5)	29(82.9)	6(17.1)		32(91.4)	3(8.6)		33(94.3)	2(5.7)	
Yes	5(12.5)	1(20.0)	4(80.0)		1(20.0)	4(80.0)		3(60.0)	2(40.0)	
No. of year/s in farming				0.026			0.004			<0.001
1–15	25(62.5)	21(84.0)	4(16.0)		23(92.0)	2(8.0)		24(96.0)	1(4.0)	
15–30	13(32.5)	9(69.2)	4(30.8)		10(76.9)	3(23.1)		12(92.3)	1(7.7)	
>30	2(5)	0(0)	2(100.0)		0(0)	2(100.0)		0(0)	2(100.0)	

Similarly, the correlation between professional farm training and knowledge of farmers about antibiotics was statistically significant (*p* < 0.05). Farmers having professional farm training (*n* = 5) have more knowledge about antibiotic usage (*n* = 4, 80.0%) and prohibited antibiotics (*n* = 4, 80.0%), while they had comparatively less knowledge about AMR (*n* = 2, 40.0%). We have also observed that the number of years in farming has a direct relation to the knowledge about antibiotics. All farmers having more than 30 years in farming had enough knowledge, as compared to those having less experience in farming. Whereas more than 80% of the farmers had no idea about antibiotic usage, AMR, and prohibited antibiotics. Therefore, these variables have statistical significance (*p* < 0.05).

### Environmental dissemination of poultry wastes

To identify the environmental dissemination of AMR from poultry farming, we collected information about waste disposal practices. Most of the poultry farmers (85.0%) reported not having any wastewater drainage system in their farms; rather the poultry waste was being drained directly into adjacent open areas and agricultural land. Only 6 farms (15.0%) had proper drainage systems. Additionally, 24 (60.0%) farmers reported that they use poultry wastes as fertilizer, which is causing the further spread of AMR to the food system.

### Health care-seeking behavior and antibiotic use in humans (poultry farmers)

Out of the 40 participants, more than one-third (n = 15, 37.5%) used antibiotics within the last month preceding the survey (*n* = 2, 5.0%), in the last 1–3 months, while (*n* = 10, 25.0%) of the participants used antibiotics 6 months prior to the survey. Two-thirds (n = 13, 32.5%) of the participants did not remember the last intake of antibiotics. About half (*n* = 21, 52.5%) of the respondents reported self-medication of antibiotics without a physician’s prescription. Almost half of the participants (*n* = 19, 47.5%) took previously used antibiotics without consulting a physician, while (*n* = 1, 2.5%) used antibiotics after getting advice from relatives. One participant (2.5%) mentioned that he had no access to physicians, so he used antibiotics without prescription.

When participants were asked about the source of antibiotics (*n* = 30.75.0%), reported obtaining them from local pharmacies (*n* = 8, 20.0%), from leftover antibiotics at home, and (*n* = 2, 5.0%) obtained them from rural medical practitioners (unqualified doctors). Moreover (*n* = 28.70.0%) of the respondents used antibiotics for 1–3 days (*n* = 8, 20.0%), used for 4–7 days, and (*n* = 4, 10.0%) used for more than 7 days (Table 6).

**Table 6:** Pattern of antibiotics use in poultry farmers (*N* = 40).

Characteristics	Total *n* (%)
Purpose of antibiotic/s use	
Flu (common cold)	17(42.5)
Gastrointestinal infections	4(10.0)
Respiratory infections	13(32.5)
Fever	3(7.5)
Others*	3(7.5)
Physician prescription	
No	21(52.5)
Yes	19(47.5)
Reason behind self-medication	
None	19(47.5)
Not access to physician care	1(2.5)
Previous experience	19(47.5)
Advice from relatives	1(2.5)
Source of getting antibiotic/s	
Pharmacy	30(75.0)
Leftover household antibiotics	8(20.0)
Rural practitioner (Untrained doctor)	2(5.0)
Duration of antibiotic/s use	
1–3 days	28(70.0)
4–7 days	8(20.0)
>7 days	4(10.0)

*Others include skin infections (*n* = 2) and Inguinal hernia (*n* = 1).

**Figure 3:**
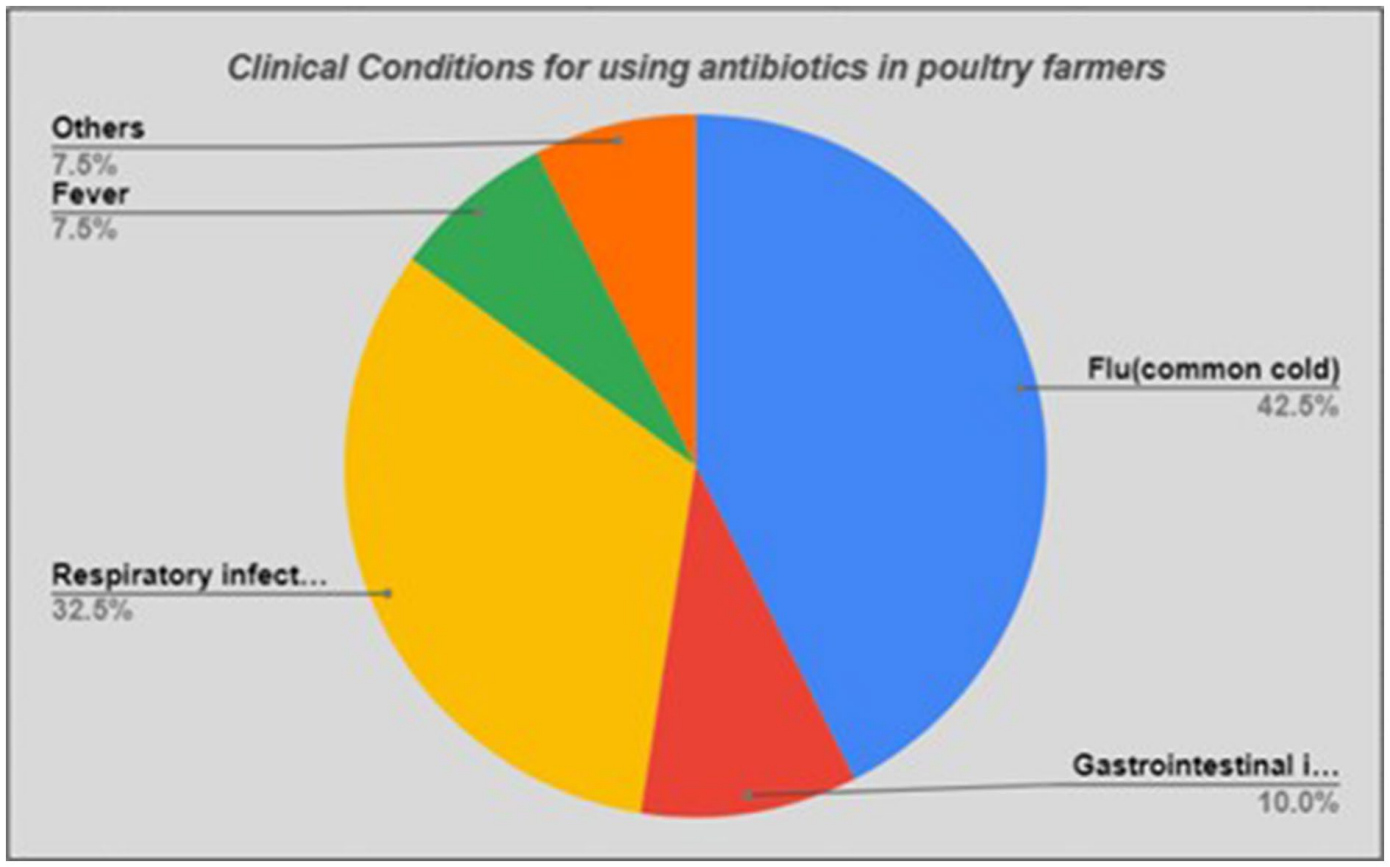
Clinical Conditions for using antibiotics in poultry farmers.

Considering the indications of antibiotic use, a large proportion (*n* = 17, 42.5%) of the participants mentioned use of antibiotics for treating flu/common cold (mostly viral), and about one-third (*n* = 13, 30.0%) stated respiratory infections in general (where cough and chest pain were common symptoms) (Figure 3).

## Discussion

In the current study, we evaluated the practice of antibiotic use and healthcare-seeking behavior regarding poultry farming and farmers in rural areas of Punjab in Pakistan which is the first of this nature in the study area. While Health care seeking behavior is defined as any action taken by an individual who identifies themselves to be ill or having health-related issues for the purpose of finding an appropriate treatment (27).

Our study findings confirm that the use of antibiotics in poultry is not well regulated in Pakistan, particularly in rural areas and it has the potential to enhance the emergence of drug resistant pathogens to develop AMR. Most of the participants used antibiotics as growth promoters without any consultation with trained veterinarians. This observation of the unregulated use of antibiotics in food-producing animals in Pakistan including improper dosage, wrong combination of antibiotics, misuse, and overuse is similar to other studies in similar settings (11, 24). In addition, we observed a significant seasonal variation in prophylactic antibiotic use in poultry. The poultry farmers use more antibiotics in winter than summer as chickens are more prone to diseases in cold weather. This information combined with an antibiotic sales report at different times, is considered crucial for a new policy and its implementation.

The majority of the participants in this study purchased antibiotics based on their previous experience and from local agents, which is a clear indication of a patron-client relationship and undue influence for unnecessary usage. Such resistance-provoking drug purchase behavior and practice is also evident in similar LMIC settings (10, 25–27). Our study participants mostly used colistin sulfate and amoxicillin trihydrate, which is alarming. Overuse and misuse of colistin lead to the development of multidrug resistance as reported in previous studies (28–30). Some farmers used antibiotics as a supplement on a daily basis while others used antibiotics on alternative days without following the duration of treatment and withdrawal time. Another important finding was the inability of the participants to distinguish between viral and bacterial infections which supports the fact that nearly half of them used antibiotics for flu (common cold) and a few used them for fever, which is supported by other studies done in Punjab and Sindh, Pakistan (31, 32). Lack of education, lack of professional farm training, and not getting advice from the veterinary doctors were the common reasons behind such misuse and these findings are in line with previous studies (11, 26, 33).

Another important concern of antibiotic use in poultry farming is the ‘Withdrawal period’. Any medicine or antibiotic consumed by humans or animals, has a withdrawal period when they become non-functional and eliminated from the body. The ‘Withdrawal period’ is particularly important for food animals such as poultry and cattle to ensure that no antibiotics have entered the human food chain. Unfortunately, nearly half of our participants were unaware of this term and so did not follow the recommended withdrawal period. This unhealthy practice increases the possibility of high levels of antibiotic residues in poultry meat with their detrimental health consequences. All these findings of violation of the withdrawal period for antibiotics have also been observed in other studies (25, 28).

Antibiotic use in poultry and lack of proper biosecurity practices are major concerns in the environmental dissemination of antibiotic residues and resistant bacteria which in turn act as the mixing hub of human-animal superbugs. A majority of farms in the current study had no wastewater drainage system and wastewater was simply drained into nearby agricultural land or open sites near farms. This practice increases the chances of antibiotic contamination of agricultural land through raw and untreated wastewater (16). Moreover, farmers sold poultry wastes to agricultural landowners to be used as fertilizer and more than half of the participants utilized poultry wastes as fertilizers for themselves as well. Several previous studies have revealed the linkage between antibiotic use in poultry and the development of AMR in humans and in surroundings through antibiotic residues in manure and urine (34–36).

In terms of antibiotic use among poultry farmers in the study area, the easy accessibility of antibiotics from pharmacies/drug stores without doctor’s prescriptions is an important issue. One-third of the participants obtained antibiotics from pharmacies and self-medication is a common practice. People in LMIC settings have no idea about the risk of self-medication and they purchase antibiotics from drug stores without a physician’s prescription (13, 37, 38). Another concern is the use of antibiotics from previous experience and from leftovers at the farmers’ homes. Our study participants also reported this practice. The main reason behind this was the financial constraints and traveling to the cities to seek physician’s consultation. This observation has also been reported in studies conducted in India, Malaysia, Sindh (Pakistan) and Lebanon (39–42).

Several studies have reported that patients understanding about illness and its treatment will increase their adherence to the medication (43, 44). In our findings, the drug adherence to antibiotics was not according to the instructions about the drug usage and most of the participants used antibiotics for 1–3 days. Participants were of the opinion that they need only to stop taking the medicine after they feel better. Improper consumption of antibiotics results in antibiotic resistance (45). Incomplete information about antibiotic use, getting only a few doses because of high prices, and use of left-over antibiotics at home are the reasons associated with it (13). Even from pharmacies or from rural practitioners, one can get antibiotics as a one-day treatment. However, non-adherence to the antibiotic regimen can be improved by increasing the general population knowledge and proper counseling at pharmacies and by improving pharmacist-patient interactions (45).

Knowledge about antibiotic resistance and antibiotic usage is a fundamental requirement to mitigate AMR at community level. A significant number of our study participants had no knowledge about these issues. Knowledge of the farmers about antibiotics was directly associated with their education level. Uneducated participants and those with a primary level of education had no or only a limited knowledge about antibiotic use, AMR, and the prohibited list of antibiotics in poultry as compared to those participants who had a secondary or higher level of education. These findings are consistent with other studies (13, 46, 47). Therefore, educational interventions can be effective to raise awareness, enhance knowledge about antibiotic use and changing their healthcare-seeking behavior. A good example is E-bug by public health England which is an international health education source to educate people about antibiotics, AMR, and infections (26, 48).

### Limitations

While our study focuses on an imperative aspect of AMR in rural Pakistan, it has few limitations. The findings may not be generalized to the whole country as we collected data from a sub-district in Punjab. Yet, these results provide a descriptive picture of the overall situation of antibiotic use in rural Pakistan. Moreover, the findings of this study may also be affected by recall bias to some extent as participants had to remember the use of antibiotics and they have very minimal medicine-related knowledge. However, we tried to validate the findings by collecting and inspecting the antibiotic boxes from the farms and households.

### Future directions and conclusion

Our study highlights the risks of AMR due to non-professional farming practices and its hazards to humans, animals, and the environment. It furthermore emphasizes the need for education and professional farm training for the containment of AMR in resource-deficient settings. The current study also strongly supports the alignment of food safety policy with the current AMR mitigation plan. An integrated and sustainable national AMR and food safety policy needs to be adopted with the inclusion of farmers’ education, mass awareness, organic farming, and strict antibiotic usage guidelines.

## Data availability statement

The raw data supporting the conclusions of this article will be made available by the authors, without undue reservation.

## Ethics statement

This study was reviewed and approved by The Ethics Committee at Institute of Health and Society, UiO (Application no. 8849626), Norwegian Centre for Research Data (reference no. 726029) and the Bioethics Committee of faculty of biological sciences, Quaid-i-Azam University, Islamabad, Pakistan (reference no. #BEC-FBS-QAU2021-250). The participants provided their written informed consent to participate in this study.

## Author contributions

MA: conceptualization and project administration. MA and UH: methodology and writing – original draft preparation. UH and MA: formal analysis. AK: resources. UH: data curation. EM, IG, and AK: writing – review and editing. MA, AK, and EM: supervision. All authors have read and agreed to the published version of the manuscript.

## Funding

This research was funded by the Department of Community Medicine and Global Health, Institute of Health and Society, Faculty of Medicine, University of Oslo, Norway.

## Conflict of interest

The authors declare that the research was conducted in the absence of any commercial or financial relationships that could be construed as a potential conflict of interest.

## Publisher’s note

All claims expressed in this article are solely those of the authors and do not necessarily represent those of their affiliated organizations, or those of the publisher, the editors and the reviewers. Any product that may be evaluated in this article, or claim that may be made by its manufacturer, is not guaranteed or endorsed by the publisher.
